# 

**Published:** 1914-06-27

**Authors:** 


### Gifts and Bequests.

Under the will, just proved, of a maiden lady living at St. Albans £200 has been left to the Norwood Cottage Hospital.

Mr. John Manuel, of Sheffield, retired house furnisher, an authority on fly-fishing, has left £100 each to the Sheffield Royal Infirmary, the Sheffield Royal Hospital, the Sheffield Free Hospital for Sick Children, and the Jessop Hospital for Women, Sheffield.

The Eversfield Chest Hospital for Consumption, St. Leonards-on-Sea, has received twenty-five guineas from the Fishmongers' Company and ten guineas from the Skinners' Company.

### PRESENTATION AT LIVINSGTONE COLLEGE.

On Saturday, June 13, on the occasion of "Commemoration Day" at Livingstone College, which was the twenty-first anniversary of its inception, a handsome presentation was made to Dr. and Mrs. Harford on their leaving the college after working there for the whole of its twenty-one years of existence. The presentation, which took the form of a silver rose bowl, album, and cheque for £100, was made on behalf of the past and present students, the committee, and a few other subscribers as a mark of regard.

RATES OF SUBSCRIPTION: Twelve months, 6/6; Six months, 4/-; Three months, 2/-, post free to any address in the United Kingdom. Abroad, Twelve months, 8/8; Six months, 5/-; Three months, 2/6, post free.—Address The Subscription Dept., "THE HOSPITAL," The Hospital Building, 28/29 Southampton Street Strand, London, W.C.

